# A 20-year overview of fertility preservation in boys: new insights gained through a comprehensive international survey

**DOI:** 10.1093/hropen/hoae010

**Published:** 2024-02-16

**Authors:** Kathleen Duffin, Nina Neuhaus, Claus Yding Andersen, Virginie Barraud-Lange, Aude Braye, Cristina Eguizabal, Aurélie Feraille, Jill P Ginsberg, Debra Gook, Ellen Goossens, Kirsi Jahnukainen, Yasmin Jayasinghe, Victoria Keros, Sabine Kliesch, Sheila Lane, Callista L Mulder, Kyle E Orwig, Ans M M van Pelt, Catherine Poirot, Michael P Rimmer, Nathalie Rives, Hooman Sadri-Ardekani, Myriam Safrai, Stefan Schlatt, Jan-Bernd Stukenborg, Marianne D van de Wetering, Christine Wyns, Rod T Mitchell

**Affiliations:** Department of Biomedical Sciences, University of Edinburgh, Edinburgh, UK; Centre of Reproductive Medicine and Andrology, University of Münster, Münster, Germany; Laboratory of Reproductive Biology, University Hospital of Copenhagen & Faculty of Health and Medical Sciences, University of Copenhagen, Copenhagen, Denmark; Department of Reproductive Biology CECOS, AP-HP Centre—University of Paris Cité, Cochin Hospital, Paris, France; AYA Unit, Fertility Preservation Consultation, Haematology Department, AP-HP Nord, University of Paris Cité, Saint-Louis Hospital, Paris, France; Department of Genetics, Reproduction and Development (GRAD), Biology of the Testis (BITE), Vrije Universiteit Brussel (VUB), Brussels, Belgium; Cell Therapy, Stem Cells and Tissues Group, Basque Center for Blood Transfusion and Human Tissues, Bizkaia, Spain; Biocruces Bizkaia Health Research Institute, Bizkaia, Spain; NorDIC, Team “Adrenal and Gonadal Pathophysiology”, Biology of Reproduction-CECOS Laboratory, Rouen University Hospital, Université de Rouen Normandie, Rouen, France; Division of Oncology, Children's Hospital of Philadelphia, Department of Pediatrics, University of Pennsylvania Perelman School of Medicine, Philadelphia, PA, USA; Reproductive Services/Melbourne IVF, The Royal Women’s Hospital, Parkville, VIC, Australia; Department of Obstetrics and Gynaecology, Royal Women’s Hospital, University of Melbourne, Parkville, VIC, Australia; Department of Genetics, Reproduction and Development (GRAD), Biology of the Testis (BITE), Vrije Universiteit Brussel (VUB), Brussels, Belgium; Childhood Cancer Research Unit, Department of Women’s and Children’s Health, NORDFERTIL Research Lab Stockholm, Karolinska Institutet and Karolinska University Hospital, Solna, Sweden; Division of Haematology-Oncology and Stem Cell Transplantation, New Children’s Hospital, Pediatric Research Center, Department of Pediatrics, University of Helsinki and Helsinki University Hospital, Helsinki, Finland; Department of Obstetrics and Gynaecology, Royal Women’s Hospital, University of Melbourne, Parkville, VIC, Australia; Oncofertility Program, Royal Children’s Hospital, Melbourne, VIC, Australia; Division of Gynecology and Reproduction, Department of Reproductive Medicine, Karolinska University Hospital, Stockholm, Sweden; Division of Urology, Department of Clinical Science, Intervention and Technology—CLINTEC, Karolinska Institutet, Stockholm, Sweden; Centre of Reproductive Medicine and Andrology, University of Münster, Münster, Germany; Department of Paediatric Oncology and Haematology, Children’s Hospital Oxford, Oxford University Hospitals NHS Foundation Trust, Oxford, UK; Reproductive Biology Laboratory, Center for Reproductive Medicine, Amsterdam UMC Location University of Amsterdam, Amsterdam, the Netherlands; Amsterdam Reproduction and Development Research Institute, Amsterdam, the Netherlands; Department of Obstetrics, Gynecology and Reproductive Sciences, Magee-Womens Research Institute, University of Pittsburgh School of Medicine, Pittsburgh, PA, USA; Reproductive Biology Laboratory, Center for Reproductive Medicine, Amsterdam UMC Location University of Amsterdam, Amsterdam, the Netherlands; Amsterdam Reproduction and Development Research Institute, Amsterdam, the Netherlands; Fertility Preservation Consultation, Haematology Department, AYA Unit, Saint Louis Hospital, AP-HP Médecine Sorbonne Université, Paris, France; Department of Reproductive Biology, Cochin Hospital, Paris, France; MRC Centre for Reproductive Health, Institute of Regeneration and Repair, University of Edinburgh, Edinburgh, UK; NorDIC, Team “Adrenal and Gonadal Pathophysiology”, Biology of Reproduction-CECOS Laboratory, Rouen University Hospital, Université de Rouen Normandie, Rouen, France; Department of Urology, Wake Forest University School of Medicine, Winston-Salem, NC, USA; Wake Forest Institute for Regenerative Medicine, Wake Forest University School of Medicine, Winston-Salem, NC, USA; Department of Obstetrics, Gynecology and Reproductive Sciences, Magee-Womens Research Institute, University of Pittsburgh School of Medicine, Pittsburgh, PA, USA; Sackler Faculty of Medicine, Department of Obstetrics and Gynecology, Chaim Sheba Medical Center (Tel Hashomer), Tel Aviv University, Tel Aviv, Israel; Centre of Reproductive Medicine and Andrology, University of Münster, Münster, Germany; Childhood Cancer Research Unit, Department of Women’s and Children’s Health, NORDFERTIL Research Lab Stockholm, Karolinska Institutet and Karolinska University Hospital, Solna, Sweden; Princess Maxima Center for Pediatric Oncology, Utrecht, the Netherlands; Department of Gynecology and Andrology, Cliniques Universitaires Saint-Luc, Université Catholique de Louvain, Brussels, Belgium; MRC Centre for Reproductive Health, Institute of Regeneration and Repair, University of Edinburgh, Edinburgh, UK; Royal Hospital for Children and Young People, Edinburgh, UK

**Keywords:** fertility preservation, spermatogonia, cryopreservation, testicular sperm extraction, testicular tissue, oncofertility, children, male, childhood cancer, chemotherapy

## Abstract

**STUDY QUESTION:**

Twenty years after the inception of the first fertility preservation programme for pre-pubertal boys, what are the current international practices with regard to cryopreservation of immature testicular tissue?

**SUMMARY ANSWER:**

Worldwide, testicular tissue has been cryopreserved from over 3000 boys under the age of 18 years for a variety of malignant and non-malignant indications; there is variability in practices related to eligibility, clinical assessment, storage, and funding.

**WHAT IS KNOWN ALREADY:**

For male patients receiving gonadotoxic treatment prior to puberty, testicular tissue cryopreservation may provide a method of fertility preservation. While this technique remains experimental, an increasing number of centres worldwide are cryopreserving immature testicular tissue and are approaching clinical application of methods to use this stored tissue to restore fertility. As such, standards for quality assurance and clinical care in preserving immature testicular tissue should be established.

**STUDY DESIGN, SIZE, DURATION:**

A detailed survey was sent to 17 centres within the recently established ORCHID-NET consortium, which offer testicular tissue cryopreservation to patients under the age of 18 years. The study encompassed 60 questions and remained open from 1 July to 1 November 2022.

**PARTICIPANTS/MATERIALS, SETTING, METHODS:**

Of the 17 invited centres, 16 completed the survey, with representation from Europe, Australia, and the USA. Collectively, these centres have cryopreserved testicular tissue from patients under the age of 18 years. Data are presented using descriptive analysis.

**MAIN RESULTS AND THE ROLE OF CHANCE:**

Since the establishment of the first formal fertility preservation programme for pre-pubertal males in 2002, these 16 centres have cryopreserved tissue from 3118 patients under the age of 18 years, with both malignant (60.4%) and non-malignant (39.6%) diagnoses. All centres perform unilateral biopsies, while 6/16 sometimes perform bilateral biopsies. When cryopreserving tissue, 9/16 centres preserve fragments sized ≤5 mm^3^ with the remainder preserving fragments sized 6–20 mm^3^. Dimethylsulphoxide is commonly used as a cryoprotectant, with medium supplements varying across centres. There are variations in funding source, storage duration, and follow-up practice. Research, with consent, is conducted on stored tissue in 13/16 centres.

**LIMITATIONS, REASONS FOR CAUTION:**

While this is a multi-national study, it will not encompass every centre worldwide that is cryopreserving testicular tissue from males under 18 years of age. As such, it is likely that the actual number of patients is even higher than we report. Whilst the study is likely to reflect global practice overall, it will not provide a complete picture of practices in every centre.

**WIDER IMPLICATIONS OF THE FINDINGS:**

Given the research advances, it is reasonable to suggest that cryopreserved immature testicular tissue will in the future be used clinically to restore fertility. The growing number of patients undergoing this procedure necessitates collaboration between centres to better harmonize clinical and research protocols evaluating tissue function and clinical outcomes in these patients.

**STUDY FUNDING/COMPETING INTEREST(S):**

K.D. is supported by a CRUK grant (C157/A25193). R.T.M. is supported by an UK Research and Innovation (UKRI) Future Leaders Fellowship (MR/S017151/1). The MRC Centre for Reproductive Health at the University of Edinburgh is supported by MRC (MR/N022556/1). C.L.M. is funded by Kika86 and ZonMW TAS 116003002. A.M.M.v.P. is supported by ZonMW TAS 116003002. E.G. was supported by the Research Program of the Research Foundation—Flanders (G.0109.18N), Kom op tegen Kanker, the Strategic Research Program (VUB_SRP89), and the Scientific Fund Willy Gepts. J.-B.S. is supported by the Swedish Childhood Cancer Foundation (TJ2020-0026). The work of NORDFERTIL is supported by the Swedish Childhood Cancer Foundation (PR2019-0123; PR2022-0115), the Swedish Research Council (2018-03094; 2021-02107), and the Birgitta and Carl-Axel Rydbeck’s Research Grant for Paediatric Research (2020-00348; 2021-00073; 2022-00317; 2023-00353). C.E is supported by the Health Department of the Basque Government (Grants 2019111068 and 2022111067) and Inocente Inocente Foundation (FII22/001). M.P.R. is funded by a Medical Research Council Centre for Reproductive Health Grant No: MR/N022556/1. A.F. and N.R. received support from a French national research grant PHRC No. 2008/071/HP obtained by the French Institute of Cancer and the French Healthcare Organization. K.E.O. is funded by the University of Pittsburgh Medical Center and the US National Institutes of Health HD100197. V.B-L is supported by the French National Institute of Cancer (Grant Seq21-026). Y.J. is supported by the Royal Children’s Hospital Foundation and a Medical Research Future Fund MRFAR000308. E.G., N.N., S.S., C.L.M., A.M.M.v.P., C.E., R.T.M., K.D., M.P.R. are members of COST Action CA20119 (ANDRONET) supported by COST (European Cooperation in Science and Technology). The Danish Child Cancer Foundation is also thanked for financial support (C.Y.A.). The authors declare no competing interests.

**TRIAL REGISTRATION NUMBER:**

N/A.

WHAT DOES THIS MEAN FOR PATIENTS? Before boys have gone through puberty, their testes are not able to produce sperm. This means that when these boys are receiving treatments that will affect their fertility, including several forms of childhood cancer treatment, they cannot yet freeze sperm for future use. The only option is to freeze fragments of immature testis tissue, with the aim of using this in the future to restore fertility. This experimental technique has been developing over the last 20 years, and here we present results of an international survey, summarizing the current activities in centres offering fertility preservation to pre-pubertal boys in Europe, Australia, and the USA. Our results have shown that more than 3000 boys have had testicular tissue frozen to preserve their fertility and this mostly includes children due to receive treatment for cancer or other conditions that could affect their future fertility. Practice is broadly similar across the 16 centres worldwide that offer testicular tissue cryopreservation, with some variation identified. Collaboration between the centres offering this procedure will be important for ensuring good outcomes for young males at risk of future infertility.

## Introduction

Given that the pre-pubertal testis is not yet capable of producing sperm, fertility preservation in this patient group is challenging. The first fertility preservation programme specifically for pre-pubertal boys was initiated in Brussels in 2002, offering cryopreservation of testicular tissue (Braye *et al.*, 2019). While this procedure remains experimental, with successful use of stored tissue to restore fertility in humans yet to be demonstrated, the number of specialized centres offering cryopreservation of immature human testicular tissue in Europe and the USA has steadily increased over the last 20 years.

In 2012, a survey undertaken by the ESHRE Task Force on Fertility Preservation revealed that 7 out of the 14 responding centres were offering cryopreservation of immature testicular tissue; at that point, over 260 boys and adolescents had undergone testicular tissue retrieval and storage (Picton *et al.*, 2015). In 2019, the ESHRE special interest groups (SIGs) for Andrology and Fertility Preservation distributed an extended survey to centres in Europe and the USA; responses were received from 24 sites offering testicular tissue cryopreservation (some as part of a network) and reporting on a total of 1033 patients (Goossens *et al.*, 2020). Throughout this time, there has been recognition of the need to coordinate research efforts, through networks such as GROWSPERM (www.growsperm.eu), and to increase knowledge and awareness of clinical and scientific issues associated with fertility preservation, through organizations such as ESHRE and the relevant SIGs (e.g. Fertility Preservation, Andrology, Stem Cells). In 2021, the European Union-funded PanCareLife Consortium, in collaboration with the International Late Effects of Childhood Cancer Guideline Harmonization Group, gathered a multidisciplinary panel of international experts to conduct a systematic literature review and release a consensus statement based on best available evidence; this statement recommended that testicular tissue cryopreservation should be offered to young male patients at high risk of infertility. However, it specified that this should be undertaken as part of a clinical trial or approved protocol and highlighted the need to centralize expertize and optimize protocols (Mulder *et al.*, 2021).

The systematic and comparative assessments of current clinical practices undertaken in 2012 and 2019 identified clinical and research challenges in the field (Picton *et al.*, 2015; Goossens *et al.*, 2020). The challenges include: consenting to an experimental procedure; familial expectations; potential malignant contamination; unknown effects of primary diagnosis or previous therapy on tissue quality; optimization and standardization of protocols for collection, transportation, and cryopreservation of testicular tissues; and defining relevant follow-up parameters and timelines for patients after testicular biopsy. Indeed, a survey of young male cancer patients and their families undertaken in Belgium over an 8-year period highlights the need to optimize multi-collaborative care pathways for fertility preservation in order to support patient decision-making and address expectations (Wyns *et al.*, 2015). Striving to address these challenges in a coordinated fashion, the ORCHID-NET Fertility Consortium (www.orchid-net.com) was founded in 2022 by basic and clinical researchers representing networks and centres from 12 countries. One of the first undertakings of this collaboration has been to perform a worldwide survey of current practice in the field of testicular cryopreservation.

For post-pubertal boys and adult men facing infertility due to disease or treatment, cryopreservation of sperm constitutes a validated clinical approach for fertility preservation, which should be offered as first line treatment even when few spermatozoa are present (Palermo *et al.*, 1992; Kamischke *et al.*, 2004; Rives *et al.*, 2022). However, as the pre-pubertal testis is not capable of producing mature spermatozoa, this is not an option for boys who have not yet undergone puberty. As such, there are ongoing efforts to utilize stored immature testicular tissue to develop clinically applicable methods of fertility preservation for this patient group. Current research endeavours are mainly focused on the isolation of spermatogonia for autologous germ cell transplantation (Sadri-Ardekani *et al.*, 2009; Hermann *et al.*, 2012; Serrano *et al.*, 2021), autologous testicular tissue grafting, and *in vitro* spermatogenesis (Wyns *et al.*, 2021; Tran *et al.*, 2022).

Significant progress has been made with regard to the strategy of testicular tissue autografting; in 2019, Fayomi *et al.* (2019) reported that the transplantation of immature testicular tissues from macaque monkeys led to germ cell differentiation and were able to use isolated sperm developed within the tissue fragments to inject into oocytes by ICSI, resulting in the birth of the first graft-derived baby primate (named GRADY). There has recently been a report on the first autotransplantation of adult testicular tissue into a male patient with impaired spermatogenesis (Jensen *et al.*, 2024), as per the protocol established by Fayomi *et al.* (2019). Whilst the tissue survived the procedure, there was no evidence of spermatogenesis in the transplanted tissue. However, extrapolating the negative results of this study to patients without a pre-existing spermatogenic defect should be avoided, as these disorders are unlikely to be resolved by re-transplanting cryopreserved tissue (Mitchell and Ives, 2024). In addition, previous studies suggest that human adult testis tissue survives less well after transplantation in xenograft models, compared to immature testis tissues (Geens *et al.*, 2006; Schlatt *et al.*, 2006; Hutka *et al.*, 2017). The approach of autologous tissue transplantation can still be regarded as the closest to clinical application for cryopreserved pre-pubertal testicular tissue.

Considering these highly promising advances, development of protocols for quality assurance of cryopreserved human testicular tissues needs to be considered. In particular, the quantity and integrity of spermatogonia present within cryopreserved testicular tissues are likely crucial. However, spermatogonial numbers change dynamically during development (Masliukaite *et al.*, 2016) and are reportedly highly heterogeneous among patients (Heckmann *et al.*, 2018; Stukenborg *et al.*, 2018; Valli-Pulaski *et al.*, 2019; Masliukaite *et al.*, 2023), which may be a reflection of the diverse range of indications for testicular tissue banking. Initially, testicular tissue banking was performed largely for patients prior to oncological treatments (Picton *et al.*, 2015). However, there has been an increasing proportion of patients requiring gonadotoxic treatment for non-malignant indications (e.g. sickle cell disease, thalassaemia) included in fertility preservation programmes (Goossens *et al.*, 2020). Given the relative rarity of many of these diagnoses, both benign and malignant, in childhood, the numbers of patients suffering from a given disease or exposed to a specific treatment within an individual treatment centre are relatively low. The formation of collaborative networks, such as the ORCHID-NET consortium, allows for sharing of information and expertize, thereby facilitating assessment of the effect of these individual diseases or treatments on spermatogonia. Additionally, while core outcome sets are being developed for use in adult male fertility trials (Rimmer *et al.*, 2022), no such consensus has been developed for the current and future use of cryopreserved pre-pubertal testis tissues. The development of an evidence-based, unified approach to patient assessment and follow-up will provide consistency of clinical care, whilst also harmonizing trial design and outcome reporting.

This study aimed to provide a framework regarding current clinical practice including patient inclusion criteria, logistics of tissue transportation and cryopreservation, quality assurance protocols for testicular tissue, and long-term clinical follow-up. To achieve this, the ORCHID-NET consortium has undertaken a comprehensive, international survey among experts in testicular cryopreservation. The resulting data provide the most extensive overview to date of fertility preservation in boys, 20 years after the initiation of the first programme.

## Materials and methods

Representatives from 17 centres that perform cryopreservation of immature testicular tissue in Europe, Australia, and the USA were invited to participate in an online survey; this encompassed the centres included in the survey by Goossens *et al.* (2020) plus some additional centres. Within each centre, individual recipients were identified on the basis of being centre-led or having an interest in male fertility preservation. The survey comprised 60 questions addressing the following aspects of testicular cryopreservation: patient numbers and demographics; clinical assessment; biopsy procedure; transportation, cryopreservation and analysis of biopsied material; funding and storage; and research (Supplementary Data File S1). Total patient numbers prior to the date of the first report (Picton *et al.*, 2015) were requested from each centre along with patient numbers for each subsequent year from 2016 onwards. Where patient numbers were requested, questions were formatted in free text style in order to gather absolute numbers rather than estimated ranges. No patient identifiable information was collected; therefore, formal ethical approval was not required. The survey was created and disseminated using Online Surveys software (Jisc, 2023), was piloted for usability and accuracy prior to distribution, and was open from 1 July 2022 to 30 November 2022. Results were collated in Excel and analysed in RStudio software using the tidyverse and finalfit packages, with plots generated using the packages ggplot2 and RColorBrewer (Wickham *et al.*, 2019; Harrison *et al.*, 2020; R Core Team, 2021; Neuwirth, 2022).

## Results

### Survey response

In total, the survey was completed by representatives of 16 of the 17 invited centres (94%) from 11 different countries (Supplementary Table S1). All respondents answered all questions, giving a 100% completion rate. Collectively, these centres report having cryopreserved testicular tissue from 3118 male patients under the age of 18 years, since the inception of their respective programmes.

While the total number of patients in the cohort is 3118, this denominator varies when patient numbers are presented by more specific parameters (e.g. age, diagnosis); this is because where centres did not have complete information, they reported all available data.

### Inclusion criteria and clinical assessment

The majority of centres (14/16; 87.5%) have defined criteria for inclusion of male patients in fertility preservation programmes. These criteria include thresholds for anticipated cyclophosphamide equivalent dose values (10/14; Supplementary Fig. S1), planned total body irradiation (14/14), and planned pelvic/testicular radiotherapy (14/14). Other inclusion criteria stated are myeloablative conditioning prior to haematopoietic stem cell transplantation, orchiectomy or ‘very high risk of infertility’ (not defined).

All patients undergo clinical assessment prior to testicular biopsy. Specifically, assessment of pubertal development through measurement of testicular volume and assessment of Tanner staging are undertaken in the majority of centres (12/16 and 10/16, respectively). Blood hormone levels are assessed in 12/16 centres, with 9/12 centres measuring the gonadotrophins LH and FSH, 8/12 measuring testosterone, and 5/12 performing additional hormonal assessments (inhibin B and/or anti-Müllerian hormone). Screening for blood-borne viruses (including HIV and hepatitis C) is carried out in 5/16 centres.

### Patient characteristics of existing cohort

**Figure 1.**
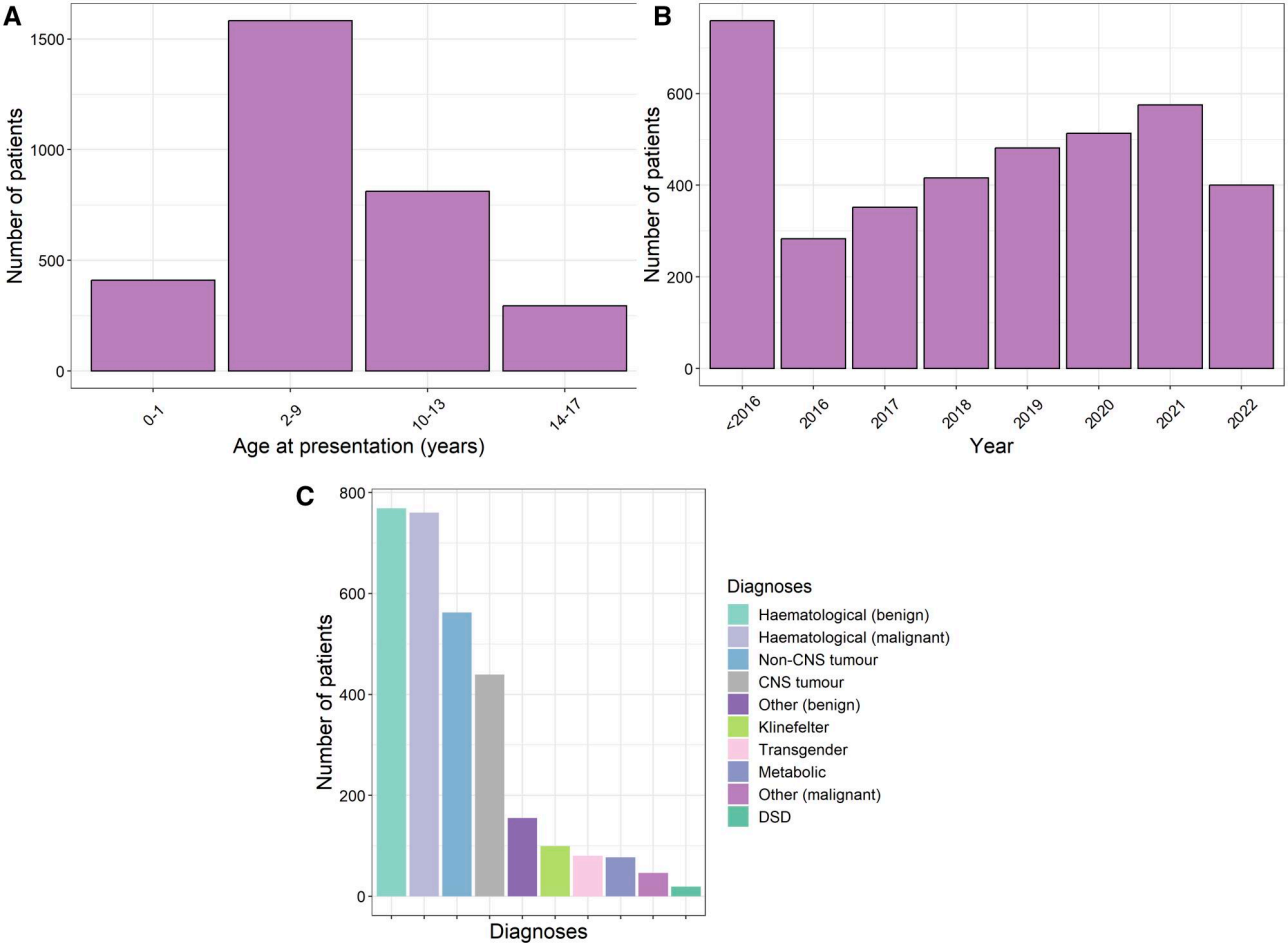
**Details of 3118 male patients who have had testicular tissue cryopreserved.** (**A**) Patients stratified by age group. (**B**) Number of patients per year. Of note, 14/16 survey responses were completed in July or August 2022; therefore, this does not represent all patients presenting in 2022. (**C**) Breakdown of diagnoses of patients undergoing testicular tissue cryopreservation. CNS: central nervous system; DSD: disorder of sex development.

The population encompasses patients under the age of 18 years (Fig. 1A). Prior to the year 2016, ∼750 patients had immature testicular tissue cryopreserved and numbers have steadily increased in each year since (Fig. 1B).

While many fertility preservation programmes were initially founded to preserve fertility of oncological patients, the majority (15/16) of centres now also offer fertility preservation to patients with non-malignant diagnoses. Within this cohort, 60.4% of patients have had testicular tissue cryopreserved due to malignant and 39.6% due to non-malignant indications. Among both categories, the most prominent patient groups are those with haematological diseases. Apart from that, diagnoses are highly heterogeneous (Fig. 1C). Although most patients have had the biopsy before starting the gonadotoxic treatment, a total of 945 patients have had biopsies performed after receiving treatment associated with low- to medium-risk gonadotoxicity, and 95 patients after receiving treatment associated with a high risk of gonadotoxicity, with fertility risk assessed on the basis of available evidence (Wyns *et al.*, 2010, 2021; Mulder *et al.*, 2021).

### Surgical procedure and transport of human testicular tissues

Regarding the surgical procedure, all centres perform unilateral biopsies and 6/16 centres sometimes perform bilateral biopsies. Half of the centres surveyed report collecting 21–30% of the testis during a unilateral biopsy (Supplementary Fig. S2A). In those patients who are in the transition phase of puberty or with established puberty, 10/16 centres combine the biopsy for preservation of spermatogonia with an initial attempt at testicular sperm extraction. Isolation of sperm is performed in theatre (3/10) or during tissue processing (7/10).

Protocols for postoperative complications are established in 12/16 centres, which report mean complication rates of 7.2% (median 1.9; range 0–70%). Specifically, wound infections were recorded in 0.7% (median 0.5%; range 0–2.6%) of all biopsied patients and bleeding requiring intervention in 0.1% (range 0–1.3%) of all biopsied patients. Of note, the upper value of 70% complication rate is significantly higher than that reported from the other centres; this particular centre reports no infection or significant bleeding but attributes the complication rate to pain exceeding that which would be associated with regular orchidopexy and requiring regular inpatient analgesia. All centres do routinely prescribe peri- and/or post-procedural analgesia, with paracetamol being the most commonly used agent (Supplementary Fig. S2B). Additional complications were haematoma, post-operative bleeding requiring compression, and pain (Supplementary Fig. S2C).

**Figure 2.**
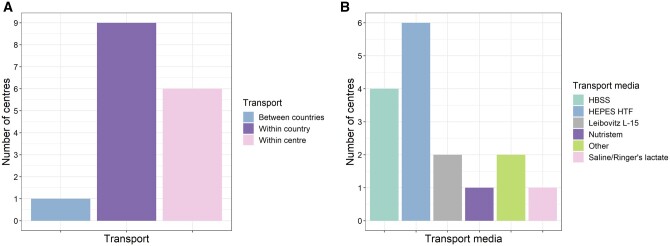
**Transport of biopsied testicular material.** (**A**) After the biopsy procedure, tissue is transported for processing and cryopreservation within and between centres and countries. (**B**) Media used for transport of biopsied tissue. HBSS: Hank’s balanced salt solution; HEPES HTF: *N*-2-hydroxyethylpiperazine-*N*′-2-ethanesulphonic acid-buffered human tubal fluid.

Following surgery, testicular tissues are transported to the site of processing and cryopreservation; in 10/16 centres, this involves transporting tissue outside the centre in which the biopsy has been taken (Fig. 2A). While centres use a variety of media for transport of tissue (Fig. 2B), the maximum time from tissue collection to tissue cryopreservation is 24 h in all centres. While material is transported at ambient temperature in 4/16 centres, the remaining 12/16 centres aim for a shipment temperature of 0–8°C. Shipment temperature is controlled in 8/16 centres, with six of these eight centres using a temperature logger for each sample.

**Figure 3.**
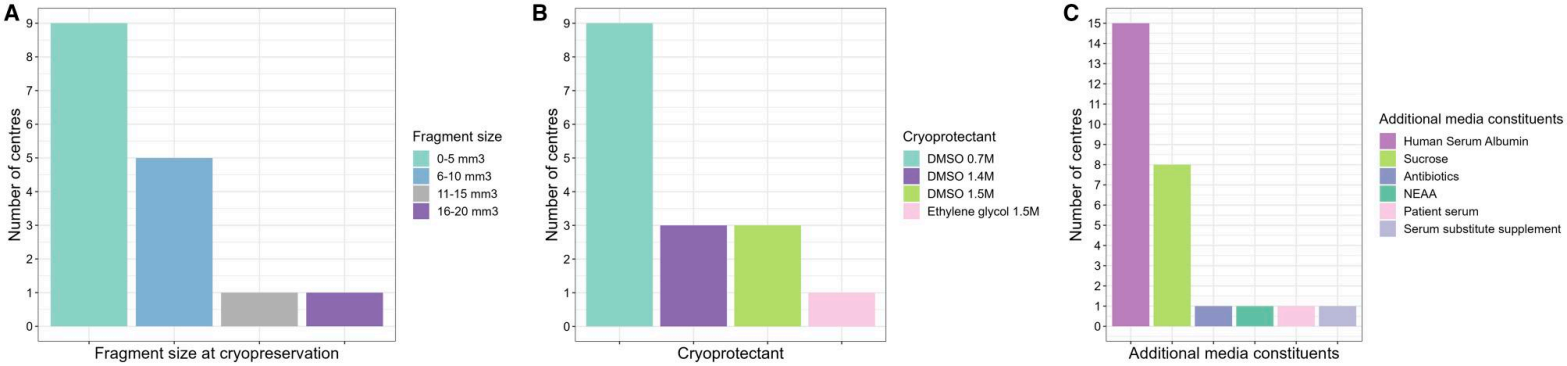
**Handling of tissue at cryopreservation centre.** (**A**) Size of testicular fragment (mm^3^) prepared for cryopreservation. (**B**) Cryoprotectants used. (**C**) Additional media constituents. All centres add additional constituents to media for cryopreservation. DMSO: dimethylsulphoxide; NEAA: non-essential amino acids.

Upon arrival, testicular tissues are cut into tissue fragments, which measure ≤5 mm^3^ in the majority of centres (Fig. 3A). Fragments are cryopreserved largely using dimethyl sulphoxide as cryoprotectant (15/16), with one centre using ethylene glycol (Fig. 3B). Additional non-permeating cryoprotectant sucrose is used by some (8/15), and medium is supplemented with additional constituents, most commonly human serum albumin (15/16 centres; Fig. 3C). Concentrations of additional media components are detailed in Supplementary Table S2. At present, there are no commercially available freezing media, which are specifically approved for cryopreservation of testicular tissue. In 11/15 centres, all components of media and cryoprotectant are clinical grade and Conformité Européenne (CE) marked; where non-CE marked products are used, this is done in accordance with local regulations and with risk assessments. One centre reports use of media components that are prepared on site. Importantly, protocols for cryopreservation of immature human testicular tissues containing spermatogonia are distinct from protocols to cryopreserve tissues containing sperm in 7/16 centres.

### Histological and immunohistochemical analyses of immature testicular tissues

**Figure 4.**
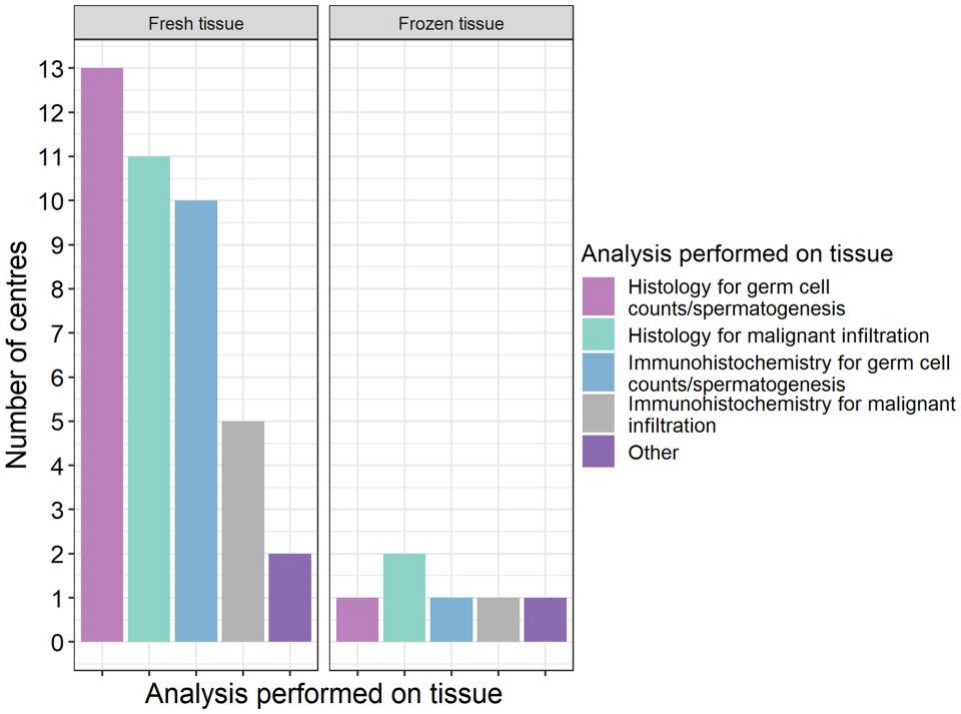
**Analysis of testicular tissues prior to and following cryopreservation and thawing.** Histological and immunohistochemical analyses conducted on testicular tissues to assess quality.

To assess the quality of testicular tissues stored for fertility preservation, a total of 15/16 centres perform histological (13/15) or immunohistochemical (10/15) analyses in order to assess germ cell counts and spermatogenesis and/or presence of malignant infiltration (Fig. 4). While these analyses are largely performed on tissue fixed at time of cryopreservation (fresh), individual centres also perform these analyses on frozen/thawed testicular tissue fragments for quality assurance purposes (Fig. 4). Immunohistochemical markers used in assessments include VASA, melanoma-associated antigen 4 (MAGE-A4), and/or Sal-like protein 4 (SALL4). Centres that perform these analyses do so on all tissues, regardless of underlying diagnosis, prior treatment, or planned treatment. The exception to this is assessment for malignant infiltration; of the 12 centres that specifically assess for this, 3 only do so in the case of a known malignant diagnosis. Relevance of routine assessment of tissues is highlighted by the finding of histological evidence of metastatic disease in the cryopreserved testicular tissue from a pre-pubertal boy with neuroblastoma, as reported in this survey and published as a case report (Ahler *et al.*, 2023).

### Funding and storage of immature human testicular tissues

**Figure 5.**
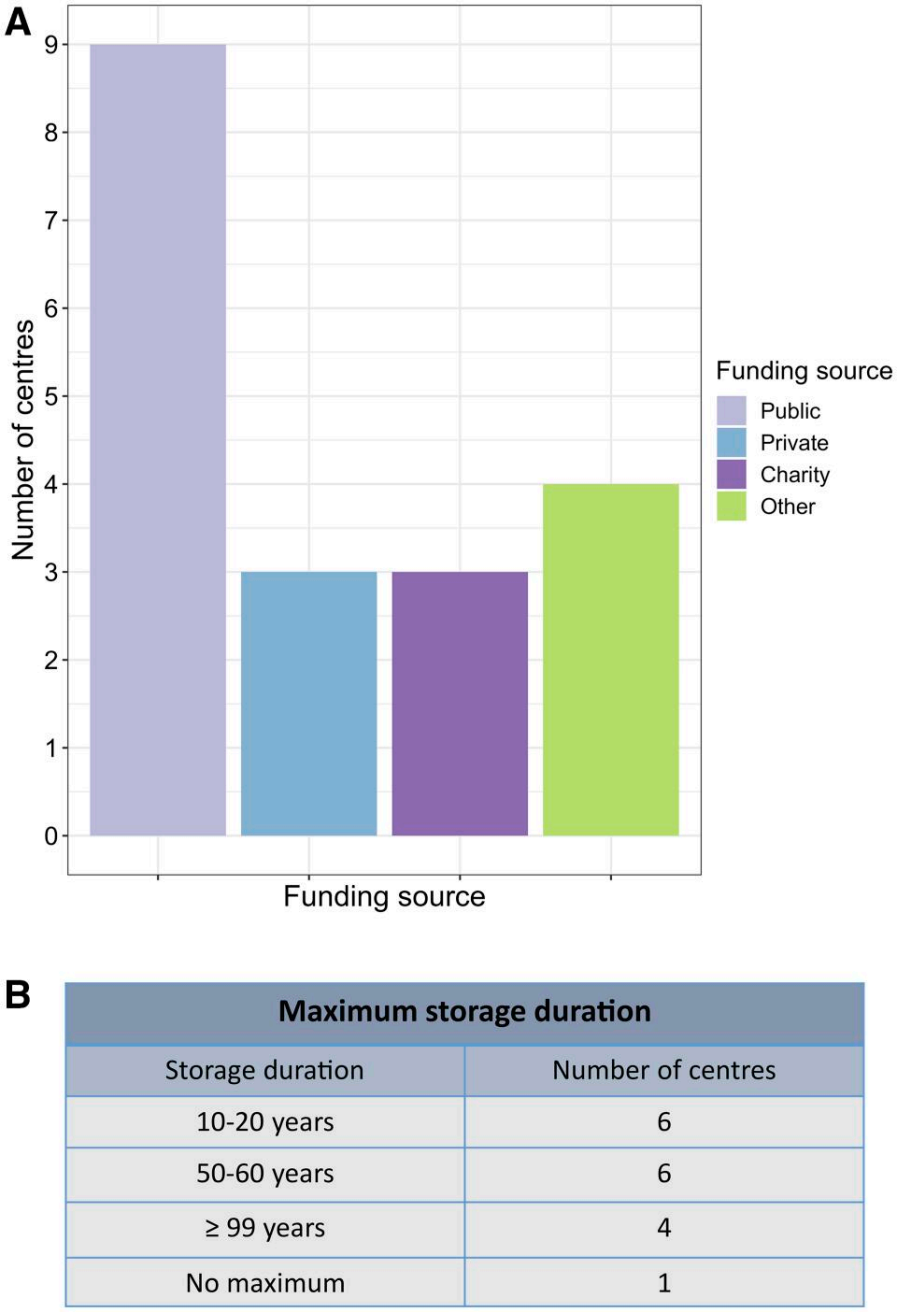
**Sources of funding and duration of tissue storage offered by centre.** (**A**) Sources of funding for testicular tissue cryopreservation. (**B**) Maximum number of years for which testicular tissue can be stored.

Internationally, there are discrepancies in how fertility preservation is funded. The costs of the surgical procedure, tissue processing, and tissue storage are not generally covered at present by insurance companies. While some centres have access to public funding, other funding sources currently include charities, research funds, institutional funds, and private funding (Fig. 5A). Furthermore, the maximum storage duration is not uniformly defined among offering centres, with a range in maximum storage duration offered from 10 to 100 years (Fig. 5B).

### Patient reproductive follow-up

For clinical follow-up of patients included in fertility preservation programmes, 10/16 centres have an established >12 monthly follow-up frequency. Clinical follow-up includes endocrinological evaluation by blood samples (8/10) for LH and FSH (7/8), testosterone (8/8), and inhibin B (6/8). Seven centres undertake semen analyses as part of routine follow-up. The timing of semen analysis varies, with 2/7 centres waiting until the patient completes puberty and 5/7 centres applying a minimum age of 16 years or 18 years (3/7 and 2/7, respectively). Given the relatively young age of this patient cohort, consent for tissue sampling and storage is typically initially obtained from parents/carers; 13 centres routinely re-consent patients when they reach adulthood. All centres give patients the opportunity to discard tissue or donate it to research if they feel it is no longer required for clinical use and would offer this choice to families if the patient dies.

### Research on immature testicular tissues

In 14/16 centres, research is conducted on small parts of the biopsied testicular tissue, with specific consent. In the event of the patient’s death, three centres dispose of the tissue and the remaining 13 centres make the tissue available for research (with 10 centres having obtained prior consent). Research efforts largely focus on grafting of testicular tissue fragments (9/13) and *in vitro* spermatogenesis (12/13). Half of the responding centres report being part of a wider clinical/research network prior to the formation of the ORCHID-NET consortium, with two of these networks spanning multiple nations.

### Transplantation of immature testicular tissue

While no centres have yet reported on transplantation of immature testicular tissue in human patients, 11 centres are planning to transplant tissue. Of these centres, four currently have ethical approval in place. Seven centres have protocols for tissue thawing and five for tissue transplantation. Of note, several survey respondents comment that they plan to request ethical approval and develop protocols in collaboration with other centres within ORCHID-NET.

## Discussion

This study has been undertaken as an initial action of the newly established ORCHID-NET consortium, aiming to assess current clinical practices in fertility preservation programmes for boys. The comprehensive survey was completed by representatives of specialized centres and fertility preservation networks offering cryopreservation of immature human testicular tissues and therefore provides an overview of current clinical practice across the world.

It is noteworthy that the number of cryopreserved testicular tissues per year has steadily increased since 2002, with limited impact of the coronavirus disease 2019 pandemic demonstrated. Total numbers of cryopreserved tissues increased from 266 in 2012 (Picton *et al.*, 2015) to 1033 in 2019 (Goossens *et al.*, 2020) and >3000 samples in 2022. This development is indicative of an increase in the number of centres reporting, as well as an increased awareness of treating physicians and affected families of this hitherto experimental approach of fertility preservation. Of note, while the total number of centres reported in this survey is lower than that in 2020 (Goossens *et al.*, 2020), that largely represents centres combining to report data centrally or as part of a network; thus, this survey reports data from centres that previously responded (Goossens *et al.*, 2020), plus some additional contributors.

Indications for testicular tissue banking remain diverse. Based on the survey by Picton *et al.* (2015), initial indications were largely focused on malignant diseases while also including some non-malignant diseases such as thalassemia. In the previous survey reported in 2020, there was wide variability between centres in the proportion of patients with non-malignant indications for fertility preservation, ranging from 2 to 98% (Goossens *et al.*, 2020); non-malignant conditions now constitute about one-third of the 3000 tissues that have been cryopreserved to date, including a minority of transgender, Klinefelter syndrome, and disorder of sex development cases. According to this study, haematological diseases constitute the dominant indication among the malignant as well as the non-malignant patient groups; this is likely due to the significantly gonadotoxic effect of haematopoietic stem cell transplantation.

However, cryopreservation in the context of malignant haematological diseases raises the issue of potential malignant contamination of testicular tissue through haematological spread, and the associated future concern of potential re-transplantation of contaminated tissue. A recent comprehensive review found overall malignant contamination rates of 37% in the pre-pubertal testis and 12% in the pre-pubertal ovary, in cases of leukaemia and lymphoma (Kourta *et al.*, 2023). This concern of malignant contamination, however, is not limited to patients with haematological malignancies; in our survey, we had one centre reporting histological evidence of metastatic neuroblastoma in otherwise normal testicular tissue cryopreserved from a pre-pubertal boy. This important finding has recently been published as a case report (Ahler *et al.*, 2023). This underscores the need to optimize protocols for assessment of tissue for malignant infiltration before re-transplantation can be considered. Given that cryopreservation and autotransplantation of ovarian tissue is increasingly well-established, it may be prudent to draw on the female experience to guide practice and research. There have been no reported cases of ovarian transplant-related cancer relapse (Sørensen *et al.*, 2014; Gellert *et al.*, 2018). Indeed, a recent report summarizes the cases of six patients who have undergone ovarian autotransplantation after treatment for acute leukaemia, with successful restoration of ovarian function and no disease relapse; the authors speculate that this is because ovarian tissue was harvested after initial non-gonadotoxic treatment, when the patient was in disease remission with little/no circulating malignant cells (Sönmezer *et al.*, 2023). However, unexpected malignant contamination of ovarian tissue has been reported (for example in a patient with apparently localized Ewing’s sarcoma) (Bittinger *et al.*, 2011; Anderson *et al.*, 2017). In their recent review, Grubliauskaite *et al.* report the detection of malignant cells in ovarian tissue cryopreserved from 25 out of 115 female patients. In addition to histological and immunohistochemical techniques, these studies employed molecular approaches to assess for minimal residual disease (Grubliauskaite *et al.*, 2023). Ongoing consideration for the optimal technique(s) to assess testicular tissue is required.

With increasing numbers of banked tissues and the approach of autologous re-transplantation of testicular tissues on the verge of clinical application, the clinical challenges defined in 2020 on standardized protocols and quality assessment on testis tissue collection, transport, and cryopreservation (Goossens *et al.*, 2020) have become of increasing importance, necessitating joint efforts of the clinics and centres involved. The data presented here demonstrate that joint efforts towards standardized approaches are already apparent, for example by the established protocols for transportation of testicular tissues within or between centres of different countries. However, many aspects of this procedure, including fragment size and culture medium used for cryopreservation and transport, remain diverse.

Considering the size of tissue fragments being cryopreserved, centres report a range of sizes from 1–5 mm^3^ up to 11–20 mm^3^. Fragment size may be partially dictated by the amount of tissue available from biopsy. It is important that the fragments are small enough to allow full penetration of cryoprotectant, and large enough to contain adequate numbers of spermatogonia and supporting cells. Keros *et al.* evaluated a protocol for cryopreservation of immature testicular tissue, using sample sizes 1–3 mm × 1–5 mm × 1–7 mm, and demonstrated maintenance of structural integrity and spermatogonia when compared with fresh tissue (Keros *et al.*, 2007). Similarly, a report from eight French CECOS centres comparing pre-pubertal testicular tissue histologically pre- and post-freezing found maintenance of spermatogonial numbers and seminiferous tubule integrity in tissue frozen in 10–15 mg fragments (Rives-Feraille *et al.*, 2022). While these studies did not assess the fertilization capacity of the tissue, the non-human primate tissue fragments used by Fayomi *et al.* (2019) to ultimately yield live offspring fell within the larger size bracket (9–20 mm^3^); the graft survival and subsequent spermatogenesis suggest that cryoprotectant penetration was adequate in those tissues. In sum, while a variety of fragment sizes are reported in this survey, they remain within a range that would be expected to allow adequate penetration of cryoprotectant and likely permit graft survival.

Particular progress has been made with regard to the development of protocols for quality assurance of tissues before and after storage. The majority of centres nowadays perform histological or immunohistochemical analyses of testicular tissues prior to cryopreservation in order to assess the presence and quantity of germ cells, with a particular focus on spermatogonia. Moreover, individual centres have established these analyses also for frozen/thawed testicular tissues, for quality assurance purposes, and have published the results of these assessments (Keros *et al.*, 2005, 2007; Kvist *et al.*, 2006; Gille *et al.*, 2021; Kabiri *et al.*, 2022; Wang *et al.*, 2022). Semi-quantitative analytical techniques have been proposed as a means of standardized quality assessment of freezing techniques and integrity of frozen tissue (Rives-Feraille *et al.*, 2022). However, it should be recognized that the stored material is relatively small and frequent quality assurance during the cryopreservation period will have a significant impact on the amount of material available for future clinical application. Combining results from different fertility preservation cohorts will enable more comprehensive analysis and provide further insights with regard to precious clinical material and rare patient groups.

One crucial development in this regard has been the establishment of a Z-score for spermatogonial numbers per round seminiferous tubule (Funke *et al.*, 2021), facilitating standardized and age-independent analyses of spermatogonial numbers within testicular tissues among different research groups. This score has been successfully applied to assess the impact of sickle cell disease and respective treatments on testicular tissues cryopreserved within the Scandinavian NORDFERTIL and the German Androprotect Network (Benninghoven-Frey *et al.*, 2022). This study demonstrated the potential of applying a uniform standardized method for quality assessment of human testicular tissue samples from different fertility preservation programmes to achieve relevant patient cohort sizes.

As the initiation of fertility preservation programmes dates back 20 years, an increasing number of boys who participated at the earlier stages have undergone puberty and reached early adulthood. The first clinical long-term follow-up focusing on the reproductive health outcome of patients who underwent testicular tissue banking for fertility preservation revealed severely impaired semen parameters in 14 out of 27 men, including 29% of the cohort showing azoospermia (Kanbar *et al.*, 2021), while a further study of 12 patients demonstrated ongoing spermatogenesis in eight patients at a median of 12.3 years after treatment (Delgouffe *et al.*, 2023). While these patient numbers are still small, the increasing numbers of testicular tissue samples representing diverse patient cohorts and follow-up of patients following successful treatments will prospectively facilitate the optimization of patient inclusion and exclusion criteria. In line with this, our survey indicated that clinical protocols for the follow-up of patients have been implemented in 13 centres, with the majority performing clinical follow-up analyses >12 monthly. In addition to semen analyses, blood tests for gonadotrophin levels, testosterone, and inhibin B levels are the most frequently performed analyses.

One limitation of this study is that it does not include all centres worldwide who offer testicular tissue cryopreservation. While we endeavoured to contact centres known to offer the procedure, we acknowledge that it was not sent to representatives on all continents and that it is possible that we have not yet been able to identify all centres currently offering testicular tissue cryopreservation. As such, there are likely to be higher patient numbers worldwide than this study presents, and there may be practices that are not being represented. One of the goals of the ORCHID-NET consortium is to encourage collaboration between centres offering testicular tissue cryopreservation, large and small, worldwide.

With a view to enhance collaboration in order to improve care and patient outcomes, the ORCHID-NET consortium (https://www.orchid-net.com) was established in 2022. The broad aim of this international network of clinicians and scientists is to develop effective fertility preservation strategies for pre-pubertal males. To address the clinical challenges in this field, the consortium’s activities and goals include documenting patient numbers and characteristics, assessment of current global practice, development of evidence-based consensus guidelines, and the establishment of a patient registry. Research priorities in this area include the development of protocols to achieve the differentiation of spermatogonia into sperm via autologous germ cell transplantation and testicular tissue grafting or *in vitro* spermatogenesis. Quality assurance of generated germ cells will be scrutinized, thereby addressing also the defined research challenges (Goossens *et al.*, 2020) in the field of male fertility preservation.

As increasing numbers of centres worldwide look to establish fertility preservation programmes, the ORCHID-NET consortium aims to use their collective expertise to provide evidence-based support for the development of programmes offering immature testicular tissue cryopreservation. Many participating centres and networks already undertake training of new centres, and the availability of this training will be broadened by ORCHID-NET. Most, if not all, participating centres have defined protocols and procedures to evaluate the performance of participating laboratories. By undertaking collaborative research to establish best practice, by creating and establishing evidence-based guidelines, and through regular collection of data to continue to assess practice, the consortium aims to harmonize approaches to training and evaluation, in order to promote optimal performance by participating centres and networks. Key points and recommendations for practice going forward, based on the conclusions from this study, are summarized in Table 1.

**Table 1.** Key points and recommendations for practice for immature testicular tissue cryopreservation.

Predicted cumulative exposure of chemotherapy and radiotherapy for all patients should be recorded prior to biopsy, in order to optimize eligibility criteria for testicular tissue cryopreservation. There is currently variation in size of testicular tissue fragment being frozen. Based on data from assessments of thawed human tissue and animal transplantation data, fragments up to 20 mm^2^ can be effectively frozen. Malignant infiltration of the testicular tissue can be present, even when it is not expected based on the diagnosis. Therefore, tissue from all patients with malignant indication for cryopreservation should be assessed for the presence of malignant infiltration. It is good practice to re-consent patients when they reach adulthood and at regular intervals thereafter. Long-term reproductive follow-up of patients who have undergone testicular tissue cryopreservation is required to understand more about long-term outcomes in this cohort.

## Conclusion

Testicular tissue cryopreservation provides a potential strategy for fertility preservation for pre-pubertal boys undergoing gonadotoxic treatment. As research in this field advances, there are increasing numbers of patients with a range of malignant and non-malignant diagnoses worldwide that will have cryopreserved immature testicular tissue. Here, we present data from an international survey, demonstrating that although there are increasingly standardized elements of clinical assessment and tissue handling, there remain wide variations in certain areas, including specific medium constituents, as well as in approach to funding, storage, and patient follow-up pathways. The ORCHID-NET consortium is a newly established network, which aims to share knowledge, expertize, and data to advance research and ultimately optimize patient care.

## Supplementary Material

hoae010_Supplementary_Data

## Data Availability

The data that support the findings of this study are available from the corresponding author upon reasonable request.
