# Supplementary material for: A 20-year overview of fertility preservation in boys: new insights gained through a comprehensive international survey
Source: Hum Reprod Open. 2024 Feb 16;2024(2):hoae010. doi: 10.1093/hropen/hoae010 (PMC10914450; doi:10.1093/hropen/hoae010)
Supplement: hoae010_Supplementary_Data [file hoae010_supplementary_data.zip › Supplementary Table S2 final.docx]

| **Supplementary Table S2:** Cryoprotectants and media constituents used for cryopreservation of testicular tissue fragments. | | | | |
| --- | --- | --- | --- | --- |
| **Cryoprotectant used** | **Concentration** | **Additional constituents** | **Concentration** | **Number of centres** |
| DMSO | 0.7M | Human Serum Albumin | 5% | 4 |
| DMSO | 0.7M | Serum substitute supplement | 5% | 1 |
| DMSO | 1.4M | Human Serum Albumin | 5% | 1 |
| DMSO | 0.7M | Human Serum Albumin | 10 mg/mL | 4 |
|  |  | Sucrose | 0.1M |  |
| DMSO | 1.4M | Human Serum Albumin | 10% | 1 |
|  |  | Sucrose | 0.15M |  |
| DMSO | 1.5M | Human Serum Albumin | 10mg/ml | 1 |
|  |  | Sucrose | 0.1M |  |
| DMSO | 1.5M | Human Serum Albumin | 10% | 2 |
|  |  | Sucrose | 0.05M |  |
|  |  | Patient serum | 10% |  |
| Ethylene Glycol | 1.5 M | Human Serum Albumin | 10 mg/ml | 1 |
|  |  | Sucrose | 0.1M |  |
| DMSO | 1.4 M | Human Serum Albumin | 10% | 1 |
|  |  | Antibiotics (e.g. penicillin/streptomycin) | 2% |  |
|  |  | NEAA | 2% |  |

DMSO: Dimethylsulphoxide.

NEAA: non-essential amino acids.
