# Supplementary material for: A 20-year overview of fertility preservation in boys: new insights gained through a comprehensive international survey
Source: Hum Reprod Open. 2024 Feb 16;2024(2):hoae010. doi: 10.1093/hropen/hoae010 (PMC10914450; doi:10.1093/hropen/hoae010)
Supplement: hoae010_Supplementary_Data [file hoae010_supplementary_data.zip › Supplementary Table S1 final.docx]

| **Supplementary Table S1:** List of participating centres who submitted data on fertility preservation in boys. | | | |
| --- | --- | --- | --- |
| **Institution** | **Department** | **City, Country** | **Network** |
| RCH & RWH Hospitals |  | Melbourne, Australia |  |
| Cliniques Universitaires Saint Luc |  | Brussels, Belgium |  |
| Vrije Universiteit Brussel and Universitair Ziekenhuis Brussel | Biology of the Testis Laboratory  Brussels IVF | Brussels, Belgium |  |
| University Hospital of Copenhagen |  | Copenhagen, Denmark |  |
| 2AP-HP.Center-University Paris Cite. Cochin Hospital. | Department of Reproductive Biology | Paris, France | CECOS |
| Charles Nicolle University Hospital Rouen | Reproductive Biology Laboratory | Rouen, France | CECOS |
| Wilhelms-Universität Münster | Centre of Reproductive Medicine and Andrology | Münster, Germany | Androprotect |
| Amsterdam UMC | Reproductive Biology Laboratory, Center for Reproductive Medicine | Amsterdam, Netherlands |  |
| Prinses Maxima Centre for Pediatric Oncology |  | Utrecht, Netherlands |  |
| Basque Center for Blood Transfusion and Human Tissues-Biocruces Bizkaia Health Research Institute |  | Galdakao, Bizkaia, Spain |  |
| Karolinska Institutet |  | Stockholm, Sweden | NORDFERTIL |
| Royal Hospital for Children and Young People |  | Edinburgh, U.K. | Edinfertility |
| Oxford University Hospitals NHS Foundation Trust | Future Fertility Programme | Oxford, U.K. |  |
| Children's Hospital of Philadelphia | Department of Pediatrics, Division of Oncology | Philadelphia, U.S.A. |  |
| University of Pittsburgh/Magee-Womens Research Institute | Center for Reproduction and Transplantation | Pittsburgh, U.S.A. |  |
| Wake Forest School of Medicine |  | Winston-Salem, U.S.A. |  |
