# Supplementary material for: A 20-year overview of fertility preservation in boys: new insights gained through a comprehensive international survey
Source: Hum Reprod Open. 2024 Feb 16;2024(2):hoae010. doi: 10.1093/hropen/hoae010 (PMC10914450; doi:10.1093/hropen/hoae010)
Supplement: hoae010_Supplementary_Data [file hoae010_supplementary_data.zip › Duffin_Supplementary_Data_File_S1.pdf]

Testicular Cryopreservation Survey - ORCHID-NET-2

Page 1

Details of person completing this form

Name of person completing this questionnaire ✱ *Required*

E-mail address of the person completing this questionnaire

Host institution of the person completing this questionnaire ✱ *Required*

Patients

In total, how many boys (<18yrs) underwent testicular tissue retrieval and cryopreservation (for preservation of SSCs) as a strategy for fertility preservation in your centre? ✱ *Required*

Please enter a whole number (integer).

How many were aged between 0-1yrs ✱ *Required*

Please enter a whole number (integer).

How many were aged between 2-9yrs ✱ *Required*

Please enter a whole number (integer).

How many were aged 10-13yrs ✱ *Required*

Please enter a whole number (integer).

How many were aged 14-17yrs ✱ *Required*

Please enter a whole number (integer).

How many patients had tissue stored prior to 2016? ✱ *Required*

More info

Please enter a whole number (integer).

How many patients had tissue stored in 2016 ✱ *Required*

More info

Please enter a whole number (integer).

How many patients had tissue stored in 2017 ✱ *Required*

More info

Please enter a whole number (integer).

How many patients had tissue stored in 2018 ✱ *Required*

More info

Please enter a whole number (integer).

How many patients had tissue stored in 2019 ✱ *Required*

More info

Please enter a whole number (integer).

How many patients had tissue stored in 2020 ✱ *Required*

More info

Please enter a whole number (integer).

How many patients had tissue stored in 2021 ✱ *Required*

More info

Please enter a whole number (integer).

How many patients had tissue stored in 2022? ✱ *Required*

More info

Please enter a whole number (integer).

Do you have inclusion criteria based on treatment risk to fertility? ✱ *Required*

- ○ Yes
- ○ No

Is Cyclophosphamide Equivalent Dose (CED) part of your inclusion criteria? ✱ *Required*

- ○ Yes
- ○ No

If you selected yes please state CED threshold for inclusion (g/m2)

Please enter a decimal number, for example: 9.63.

Is Total Body Irradiation (TBI) part of your inclusion criteria? ✱ *Required*

- ○ Yes
- ○ No

Is direct Radiotherapy to pelvis/testis part of your inclusion criteria? ✱ *Required*

- ○ Yes
- ○ No

If you selected yes please state radiotherapy threshold for inclusion (gray)

Please enter a decimal number, for example: 9.63.

Please describe any other inclusion criteria based on risk to fertility

In total, how many patients were treated for a malignant condition? ✱ *Required*

Please enter a whole number (integer).

How many were treated for a haematological malignancy (e.g.leukamia, lymphoma)? ✱ *Required*

Please enter a whole number (integer).

How many were treated for a malignant CNS condition (e.g. brain tumour)? ✱ *Required*

Please enter a whole number (integer).

How many were treated for a solid (non-CNS) malignant condition? ✱ *Required*

Please enter a whole number (integer).

Other (please specify)? ✱ *Required*

How many patients with malignant disease underwent their biopsy prior to receiving any chemotherapy/radiotherapy? ✱ *Required*

Please enter a whole number (integer).

How many had a biopsy after initial 'low-medium' risk of gonadotoxicity but prior to relapse treatment (including HSCT/BMT)? ✱ *Required*

More info

How many had a biopsy after treatment with chemo/radiotherapy associated with 'high risk' of gonadotoxicity? ✱ *Required*

More info

Do you offer testicular cryopreservation to patients with non-malignant conditions? ✱ *Required*

- Yes
- No

In total, how many had a non-malignant condition? ✱ *Required*

How many had a non-malignant haematological condition (e.g. Sickle cell, thalassaemia, aplastic anaemia, immunodeficiency)? ✱ *Required*

How many had a metabolic condition? ✱ *Required*

How many had Klinefelter Syndrome? ✱ *Required*

How many had a DSD (excluding Klinefelter Syndrome)? ✱ *Required*

How many were Transgender? ✱ *Required*

Other (please specify) ✱ *Required*

What is the age range of patients from whom testicular tissue is stored to preserve SSCs? ✱ *Required*

- ○ 0-18yrs
- ○ 0-16yrs
- ○ 0-14yrs
- ○ Other

If you selected Other, please specify:

Is Tanner staging available prior to testicular biopsy? ✱ *Required*

- Yes
- No

Do you measure testicular volumes prior to testicular biopsy? ✱ *Required*

- Yes
- No

Do you perform blood tests prior to testicular biopsy? ✱ *Required*

- Yes
- No

Which blood tests do you routinely perform prior to testicular biopsy? ✱ *Required*

- ☐ LH/FSH
- ☐ Testosterone
- ☐ Inhibin B
- ☐ AMH
- ☐ Other

If you selected Other, please specify:

Do you always perform a unilateral orchiectomy to remove a whole testis? ✱ *Required*

- Yes
- No

Do you perform bilateral biopsies? ✱ *Required*

- Always
- Sometimes
- Never

For bilateral biopsies, what proportion of the the testis is typically biopsied? ✱ *Required*

- 0-10%
- 11-20%
- 21-30%
- 31-40%
- 41-50%
- >50%

For unilateral biopsies, what proportion of the the testis is typically biopsied? ✱
Required

- 0-10%
- 11-20%
- 21-30%
- 31-40%
- 41-50%
- >50%
- n/a

If the patient is established in puberty do you combine testicular tissue biopsy with initial attempt at testicular sperm extraction (TESE)? ✱ *Required*

- Yes
- No

For tissues that may contain sperm, do you attempt Testicular Sperm Extraction (TESE)?
✱ *Required*

- ◯ In theatre
- ◯ During tissue processing (for cryopreservation)
- ◯ Not attempted

Do you have a protocol for monitoring and recording post-operative complications? ✱ *Required*

- Yes
- No

In total, what percentage of patients have post-operative complications? ✱ *Required*

Please enter a decimal number, for example: 9.63.

What percentage of all patients who have had a biopsy have post-operative wound infection at biopsy site? ✱ *Required*

Please enter a decimal number, for example: 9.63.

What percentage of all patients who have had a biopsy have post-operative bleeding requiring intervention (e.g. surgery, transfusion)? ✱ *Required*

Please enter a decimal number, for example: 9.63.

Have you encountered any other post-operative complications? Please specify complication and frequency (% of all patients).

Which of the following peri-operative analgesia are routinely prescribed? ✱ *Required*

- [ ] Local anaesthetic in theatre
- [ ] Paracetamol
- [ ] NSAIDs
- [ ] Opiates
- [ ] Other (please specify)

If you selected Other, please specify:

Cryopreservation

Which base media are testicular tissues transported in? ✱ *Required*

- HBSS
- Leibovitz L-15
- HEPES HTF
- Saline/Ringers Lactate
- Nutristem
- Other

If you selected Other, please specify:

Do you transport clinical biospy material? ✱ *Required*

- Within a single centre only
- Between centres in the same country
- Between centres in different countries

What is the maximum time allowed from tissue collection to cryopreservation? ✱ *Required*

- within 24hrs
- within 48hrs
- within 72hrs
- 72hrs or more

What temperature range is the tissue transported at? ✱ *Required*

- ○ 0-8 oC
- ○ Ambient temperature
- ○ Other

If you selected Other, please specify:

[ ]

Is transport temperature controlled? ✱ *Required*

- ○ Yes
- ○ No

How is temperature controlled?

[ ]

Is transport temperature recorded with a temperature logger for each sample? ✱ *Required*

- ○ Yes
- ○ No

What are the approximate sizes (mm^3) of the testicular fragments at cryopreservation? ✱ *Required*

- ◌ 0-5 mm3
- ◌ 6-10 mm3
- ◌ 11-15 mm3
- ◌ 16-20 mm3
- ◌ >20 mm3

Which cryoprotectants are used to freeze testicular tissues? ✱ *Required*

[ ]

What concentration of the selected cryoprotectant do you use? ✱ *Required*

[ ]

If you selected Other, please specify:

[ ]

What additional constituents are added to the media?

- ☐ Human Serum Albumin
- ☐ Serum substitute supplement
- ☐ Sucrose
- ☐ Patient serum
- ☐ Antibiotics (e.g. penicillin/streptomycin)
- ☐ Other

List the concentrations used for each media constituent. ✱ *Required*

If you selected Other, please specify:

Are all components of the media/cryoprotectant clinical grade and CE marked? ✱ *Required*

- Yes
- No

Please list any non-CE marked media components

Do you have a separate protocol for cryopreserving tissue that may contain sperm? ✱ *Required*

- Yes
- No

What are the differences in the process for collection and freezing tissues that may contain sperm? ✱ *Required*

- ☐ Collection of tissue e.g. separate biopsy for SSC or sperm collection/storage
- ☐ Transport media
- ☐ Transport temperature
- ☐ Cryoprotectant
- ☐ Preparation of tissue
- ☐ Freezing protocol

For each aspect, please describe the difference in processes for tissues that may contain sperm.

Clinical Assessment

Do you perform pathology (histology/immunohistochemistry) of **freshly fixed** tissue for clinical purposes after retrieval? ✱ *Required*

- Yes
- No

What pathological analysis do you perform (indicate all that apply)? ✱ *Required*

- [ ] Histology for malignant infiltration
- [ ] Immunohistochemistry for malignant infiltration
- [ ] Histology for germ cell counts/spermatogenesis
- [ ] Immunohistochemistry for germ cell counts/spermatogenesis
- [ ] Other

If you selected Other, please specify:

Do you perform analysis (histology/immunohistochemistry) of **frozen/thawed** material for clinical purposes shortly after freezing? ✱ *Required*

- ○ Yes
- ○ No

What pathological analysis do you perform (indicate all that apply)? ✱ *Required*

- ☐ Histology for malignant infiltration
- ☐ Immunohistochemistry for malignant infiltration
- ☐ Histology for germ cell counts/spermatogenesis
- ☐ Immunohistochemistry for germ cell counts/spermatogenesis
- ☐ Other

If you selected Other, please specify:

Do you discuss results of pathology with the patient/family ✱ *Required*

- Always
- Sometimes
- Never

Do you routinely follow-up testicular function or fertility status after biopsy? ✱ *Required*

- Yes
- No

How often do you perform follow-up of testicular function after biopsy? ✱ *Required*

- 1-3 monthly
- 4-6 monthly
- 7-9 monthly
- 10-12 monthly
- >12 monthly

Do you routinely perform blood tests following testicular biopsy? ✱ *Required*

- ○ Yes
- ○ No

Which blood tests do you routinely perform following a testicular biopsy? ✱ *Required*

- ☐ LH/FSH
- ☐ Testosterone
- ☐ Inhibin B
- ☐ Other

If you selected Other, please specify:

Do you routinely offer semen analysis to patients who have previously had a testicular biopsy? ✱ *Required*

- ◯ Yes
- ◯ No

At what age do you routinely offer semen analysis following testicular biopsy? ✱
Required

- 16
- 18
- Other

If you selected Other, please specify:

Funding and storage

How is your testicular tissue cryopreservation programme funded? ✱ *Required*

- ☐ Health Service (public funding)
- ☐ Private (e.g. medical insurance, patient)
- ☐ Charity
- ☐ Other

If you selected Other, please specify:

What is the fate of the tissue in the event of the patients death? ✱ *Required*

- ○ Tissue is disposed of
- ○ Tissue is made available for research (with prior consent)
- ○ Tissue is made available for research (without prior consent)
- ○ Other

If you selected Other, please specify:

What is the maximum duration (years) for tissue storage? ✱ *Required*

Please enter a whole number (integer).

Research

Do you conduct ethically approved research with a portion of the material collected at biopsy? ✱ *Required*

- ◯ Yes
- ◯ No

What proportion of the biospy is allocated to research? ✱ *Required*

- <10%
- <20%
- <30%
- <40%
- <50%
- >50%

Does your research involve transplantation of biopsy material into animals? ✱ *Required*

- Yes
- No

Does your research involve using of biopsy material to develop in-vitro gametogenesis? ✱ *Required*

- Yes
- No

Is your group part of a wider clinical/research network? ✱ *Required*

- Yes
- No

Name of your research network ✱ *Required*

List participating countries in your research network ✱ *Required*

List participating centres in your research network ✱ *Required*

Please provide any additional comments about your testicular tissue cryopreservation programme ✱ *Required*

Final page

Thank you for taking the time to complete the survey.

Key for selection options

33 - Which cryoprotectants are used to freeze testicular tissues?

DMSO
Ethylene Glycol
Other
